# Supplementary material for: Reproducible, portable, and efficient ancient genome reconstruction with nf-core/eager
Source: PeerJ. 2021 Mar 16;9:e10947. doi: 10.7717/peerj.10947 (PMC7977378; doi:10.7717/peerj.10947)
Supplement: Supplemental Information 1 — Markdown walkthroughs on benchmarking environments were set up and pipeline comparisons carried out, as well as raw results files and R notebooks. [file peerj-09-10947-s001.zip › supplement/benchmarking/benchmarking_runtime_parsing.html]

nf-core/eager Runtime Benchmarking Comparisons


# nf-core/eager Runtime Benchmarking Comparisons

#### James A. Fellows Yates

## Notebook Setup

This notebook will use the `tidyverse` set of packages for data loading, manipulation and plotting, and `knitr` for easy printing of markdown tables for the main paper.

```
library(tidyverse)
```

```
## ── Attaching packages ──────────────────────────────────────────────────────────────────────────────────────────────────────────────────────────────── tidyverse 1.3.0 ──
```

```
## ✓ ggplot2 3.3.2     ✓ purrr   0.3.4
## ✓ tibble  3.0.3     ✓ dplyr   1.0.1
## ✓ tidyr   1.1.1     ✓ stringr 1.4.0
## ✓ readr   1.3.1     ✓ forcats 0.5.0
```

```
## ── Conflicts ─────────────────────────────────────────────────────────────────────────────────────────────────────────────────────────────────── tidyverse_conflicts() ──
## x dplyr::filter() masks stats::filter()
## x dplyr::lag()    masks stats::lag()
```

```
library(knitr)

sessionInfo()
```

```
## R version 3.6.3 (2020-02-29)
## Platform: x86_64-pc-linux-gnu (64-bit)
## Running under: Ubuntu 20.04.1 LTS
## 
## Matrix products: default
## BLAS:   /usr/lib/x86_64-linux-gnu/blas/libblas.so.3.9.0
## LAPACK: /usr/lib/x86_64-linux-gnu/lapack/liblapack.so.3.9.0
## 
## locale:
##  [1] LC_CTYPE=en_GB.UTF-8       LC_NUMERIC=C              
##  [3] LC_TIME=en_GB.UTF-8        LC_COLLATE=en_GB.UTF-8    
##  [5] LC_MONETARY=en_GB.UTF-8    LC_MESSAGES=en_GB.UTF-8   
##  [7] LC_PAPER=en_GB.UTF-8       LC_NAME=C                 
##  [9] LC_ADDRESS=C               LC_TELEPHONE=C            
## [11] LC_MEASUREMENT=en_GB.UTF-8 LC_IDENTIFICATION=C       
## 
## attached base packages:
## [1] stats     graphics  grDevices utils     datasets  methods   base     
## 
## other attached packages:
##  [1] knitr_1.29      forcats_0.5.0   stringr_1.4.0   dplyr_1.0.1    
##  [5] purrr_0.3.4     readr_1.3.1     tidyr_1.1.1     tibble_3.0.3   
##  [9] ggplot2_3.3.2   tidyverse_1.3.0
## 
## loaded via a namespace (and not attached):
##  [1] Rcpp_1.0.5       cellranger_1.1.0 pillar_1.4.6     compiler_3.6.3  
##  [5] dbplyr_1.4.4     tools_3.6.3      digest_0.6.25    lubridate_1.7.9 
##  [9] jsonlite_1.7.0   evaluate_0.14    lifecycle_0.2.0  gtable_0.3.0    
## [13] pkgconfig_2.0.3  rlang_0.4.7      reprex_0.3.0     cli_2.0.2       
## [17] rstudioapi_0.11  DBI_1.1.0        yaml_2.2.1       haven_2.3.1     
## [21] xfun_0.16        withr_2.2.0      xml2_1.3.2       httr_1.4.2      
## [25] fs_1.5.0         hms_0.5.3        generics_0.0.2   vctrs_0.3.2     
## [29] grid_3.6.3       tidyselect_1.1.0 glue_1.4.1       R6_2.4.1        
## [33] fansi_0.4.1      readxl_1.3.1     rmarkdown_2.3    modelr_0.1.8    
## [37] blob_1.2.1       magrittr_1.5     backports_1.1.8  scales_1.1.1    
## [41] ellipsis_0.3.1   htmltools_0.5.0  rvest_0.3.6      assertthat_0.2.1
## [45] colorspace_1.4-1 stringi_1.4.6    munsell_0.5.0    broom_0.7.0     
## [49] crayon_1.3.4
```

## Data Loading

We will load the pre-aggregated and downloaded runtimes as recorded by the GNU `time` unix utility

```
results <- read_tsv("benchmarking_aggregated_runtimes.txt", 
                    col_names = c("Run", "Runtime"))
```

```
## Parsed with column specification:
## cols(
##   Run = col_character(),
##   Runtime = col_character()
## )
```

## Data Cleaning

Next we can clean up the file names to find the corresponding pipeline name.

```
results_clean <- results %>%
  separate(col = Run, sep = ":", c("File", "Line", "Category")) %>%
  select(-Line) %>%
  mutate(
    File = str_remove(File, "time_") %>%
      str_remove(".log") %>%
      str_remove("runtimes/") %>%
      str_replace("nf-core_eager", "nf-core/eager") %>%
      str_replace("paleomix_optimised", "paleomix-optimised"),
    Runtime_Minutes = map(Runtime, ~ str_split(.x, "m") %>%
      unlist() %>%
      unlist() %>%
      pluck(1)) %>% unlist() %>% as.numeric()
  ) %>%
  separate(File, sep = "_", into = c("Pipeline", "Replicate")) %>%
  select(-Runtime) %>%
  filter(Replicate != 1)
```

## Data Summaries

To get the final results we will summarise the mean and standard deviation of the three runtime metrics and print the table as markdown.

```
results_final_tidy <- results_clean %>%
  group_by(Pipeline, Category) %>%
  summarise(
    Mean = round(mean(Runtime_Minutes), digits = 1),
    SD = round(sd(Runtime_Minutes), digits = 1)
  ) %>%
  arrange(Category, Mean)
```

```
## `summarise()` regrouping output by 'Pipeline' (override with `.groups` argument)
```

```
results_final_print <- results_final_tidy %>%
  unite(col = "Mean Runtime", Mean, SD, sep = " ± ") %>%
  pivot_wider(names_from = Category, values_from = `Mean Runtime`) %>%
  kable()

results_final_print
```

| Pipeline | real | sys | user |
| --- | --- | --- | --- |
| nf-core-eager-optimised | 105.6 ± 4.6 | 13.6 ± 0.7 | 1593 ± 79.7 |
| paleomix-optimised | 130.6 ± 8.7 | 12 ± 0.7 | 1820.2 ± 36.9 |
| nf-core-eager | 209.2 ± 4.4 | 11 ± 0.9 | 1407.7 ± 30.2 |
| EAGER | 224.2 ± 4.9 | 22.9 ± 0.3 | 1736.3 ± 70.2 |
| paleomix | 314.6 ± 2.9 | 10.7 ± 1 | 1506.7 ± 14 |

## Data Plotting

We can also plot the results.

```
## Get get order of fastest to slowest based on real time
results_to_plot <- results_clean %>% mutate(Pipeline = factor(Pipeline, levels = rev(results_final_tidy$Pipeline %>% unique)))

ggplot(results_to_plot, aes(Runtime_Minutes, Pipeline)) +
  geom_violin(aes(colour = Pipeline)) +
  geom_point(pch = 20, alpha = 0.7) +
  xlab("Runtime (minutes)") +
  facet_wrap(~Category, scales = "free_x") +
  scale_colour_brewer(palette = "Set1", guide = guide_legend(nrow = 2)) +
  theme_minimal() +
  theme(legend.position = "bottom")
```
