# Supplementary figures and images for: Reproducible, portable, and efficient ancient genome reconstruction with nf-core/eager
Source: PeerJ. 2021 Mar 16;9:e10947. doi: 10.7717/peerj.10947 (PMC7977378; doi:10.7717/peerj.10947)

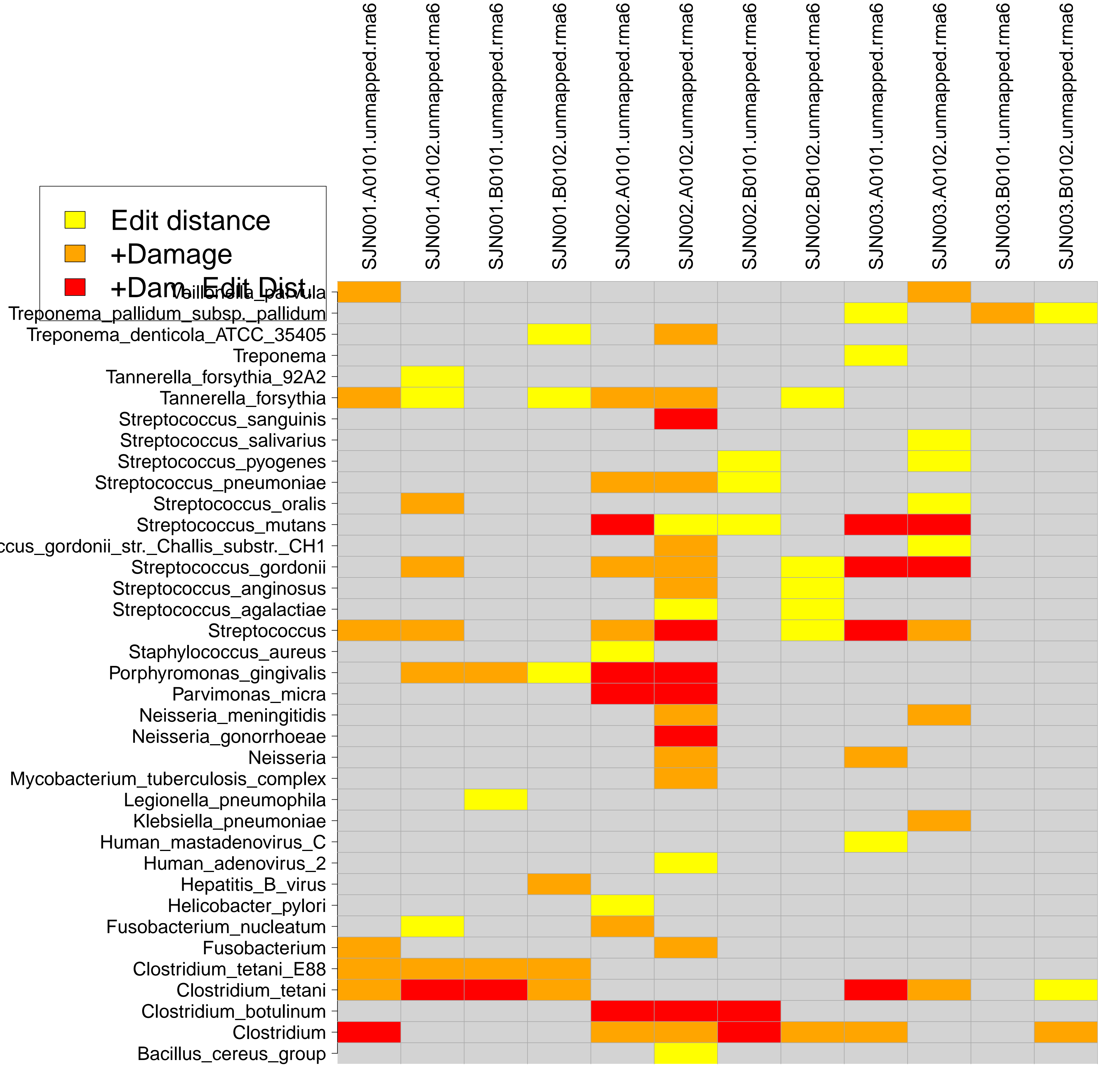

Supplement: Supplemental Information 1 — Markdown walkthroughs on benchmarking environments were set up and pipeline comparisons carried out, as well as raw results files and R notebooks. [file peerj-09-10947-s001.zip › supplement/demonstration/heatmap_overview_Wevid.pdf]
